# Road map for the clinical application of the basophil activation test in food allergy

**DOI:** 10.1111/cea.12964

**Published:** 2017-08-01

**Authors:** A. F. Santos, W. G. Shreffler

**Affiliations:** ^1^ Department of Paediatric Allergy King's College London/Guy's and St Thomas' Hospital London UK; ^2^ Department of Pediatrics Division of Allergy and Immunology Food Allergy Center Massachusetts General Hospital Harvard Medical School Boston MA USA

## Abstract

The diagnosis of IgE‐mediated food allergy based solely on the clinical history and the documentation of specific IgE to whole allergen extract or single allergens is often ambiguous, requiring oral food challenges (OFCs), with the attendant risk and inconvenience to the patient, to confirm the diagnosis of food allergy. This is a considerable proportion of patients assessed in allergy clinics. The basophil activation test (BAT) has emerged as having superior specificity and comparable sensitivity to diagnose food allergy, when compared with skin prick test and specific IgE. BAT, therefore, may reduce the number of OFC required for accurate diagnosis, particularly positive OFC. BAT can also be used to monitor resolution of food allergy and the clinical response to immunomodulatory treatments. Given the practicalities involved in the performance of BAT, we propose that it can be applied for selected cases where the history, skin prick test and/or specific IgE are not definitive for the diagnosis of food allergy. In the cases that the BAT is positive, food allergy is sufficiently confirmed without OFC; in the cases that BAT is negative or the patient has non‐responder basophils, OFC may still be indicated. However, broad clinical application of BAT demands further standardization of the laboratory procedure and of the flow cytometry data analyses, as well as clinical validation of BAT as a diagnostic test for multiple target allergens and confirmation of its feasibility and cost‐effectiveness in multiple settings.

## 1 INTRODUCTION

The prevalence of IgE‐mediated food allergy is increasing and so is the public awareness about food allergy, which together have resulted in a high demand for food allergy testing.^1^, ^2^ Following the clinical assessment of patients, which includes the clinical history and a detailed dietary history, diagnosing IgE‐mediated food allergy requires documentation of food‐specific IgE using skin prick testing (SPT) and/or specific IgE testing.^3^ However, far more common than having food allergy is to have detectable food‐specific IgE. Without a clear and recent history of an allergic reaction to the suspected food or alternatively a clear history of tolerating age‐appropriate portions of the food, the interpretation of SPT or specific IgE results can be challenging.^4^ Therefore, food allergy testing is most useful when directed from the information collected from the clinical history.^5^ Patients with equivocal history and testing should be offered an oral food challenge (OFC), the current gold standard for diagnosis.^3^, ^6^

## 2 DO WE NEED IMPROVED DIAGNOSTIC TESTING FOR IGE‐MEDIATED FOOD ALLERGY?

The diagnostic performance of SPT and specific IgE to whole extracts can vary depending on the food sources and the quality of the allergen extracts.^5^ Allergen extracts usually contain the major and minor allergens that are relevant for the ability of the food to elicit allergic reactions. However, allergen extracts obtained from certain food sources, such as soya, wheat and certain nuts and seeds, may miss some important allergens (e.g., lipophilic proteins, such as oleosins,^7^ and other proteins that are lost during the process of producing the extracts), which can impair their diagnostic utility. Generally, when interpreting SPT and specific IgE as positive at the low limits of detection, SPT and specific IgE have a high sensitivity but poor specificity. Therefore, without a clinical history that is suggestive of allergy, the mere detection of sensitization by SPT or specific IgE leads to high false‐positive rates and low positive predictive values (PPVs). When 95% PPV value cut‐offs are used (e.g., 8 mm for SPT to peanut and 15 KU/L for specific IgE to peanut^8^, ^9^), the specificity of these tests is enhanced but their sensitivity is reduced, resulting in many false negatives and low negative predictive value (NPV). Therefore, a large proportion of patients tested, particularly when the pre‐test probability is low (e.g., no or remote history of known ingestion), have intermediate range results for SPT and specific IgE and require OFC to clarify whether or not they have food allergy.^10^

These concepts also apply for specific IgE testing to individual food allergen components. The diagnostic utility of this “component testing” varies with the allergen in question. Some allergen components have shown to be more useful than the whole allergen extract in distinguishing allergic from non‐allergic patients (e.g., Ara h 2 from peanut^4^, ^11^ and Cor a 9 and Cor a 14 from hazelnut^12^, ^13^) as opposed to other components which do not seem to offer additional diagnostic accuracy compared to using whole allergen extracts (e.g., Jug r 1 in walnut allergy^14^). Other examples of components which can support food allergy diagnosis are specific IgE to Bet v 1‐homologues, such as Ara h 8 and Cor a 1, which can help to distinguish pollen‐food syndrome (e.g., secondary to birch pollen allergy) from “true” plant food allergy (e.g., systemic peanut or hazelnut allergies).^15^, ^16^, ^17^, ^18^ Specific IgE to cow's milk allergens casein, alpha‐lactalbumin and beta‐lactoglobulin and specific IgE to the egg white allergens, ovalbumin and ovomucoid, do not seem to provide additional information compared to whole allergen extracts when diagnosing cow's milk and egg allergies; however, casein and ovomucoid can be useful in identifying patients who are allergic to baked cow's milk and baked egg, respectively, as well as patients with persistent cow's milk and egg allergies.^19^, ^20^, ^21^ For the component‐specific IgE that have shown additional diagnostic value compared to specific IgE to whole extracts, their enhanced diagnostic performance usually results from higher specificity with comparable sensitivity—for example, considering the cut‐off of approximately 1 KU/L, the specificity of Ara h 2‐specific IgE was 85% (with 92% sensitivity) and the specificity of specific IgE to peanut was 38% (with 96% sensitivity) in a Swedish study.^22^ In a Dutch study, Cor a 9‐specific IgE and Cor a 14‐specific IgE had higher specificity compared to specific IgE to whole hazelnut extract.^12^ Specific IgE to allergen components are now available from several foods and can be requested in addition or instead of specific IgE to allergen extracts. The results of specific IgE to allergen components need nevertheless to be interpreted in the light of the clinical history and even those components associated with the best test performance may not confirm or exclude food allergy with high enough certainty to forgo OFC in many cases. For instance, in the Healthnuts study, 18% of infants with specific IgE to Ara h 2 lower than 0.35 KU/L reacted to peanut during the OFC, 5% of infants with specific IgE greater or equal to 1 KU/L passed the OFC and a significant proportion (22%) had results ranging between 0.1 and 1.0 KU/L, which were considered equivocal and an indication for OFC.^23^

In specialized clinics, typically 20%‐50%^4^, ^24^, ^25^ of patients undergoing OFC, and up to 70% in some reports,^26^ develop an allergic reaction during the OFC. The proportion of positive OFC depends on the criteria chosen to refer patients for OFC. The severity of the allergic reactions is unpredictable and, while generally regarded as a safe procedure in qualified settings, severe reactions can and do occur during OFC to different foods in patients with varying degrees of IgE sensitization.^27^ The majority of severe reactions occur when less than half of the challenge food has been ingested.^27^ Furthermore, OFCs require significant resources and a highly skilled clinical team experienced in this procedure and prepared to treat severe allergic reactions including severe anaphylaxis.

Novel approaches with the potential to improve the accuracy of existing allergy tests and reduce the need for OFC, both for diagnostic and for monitoring purposes, especially given the multiple new therapeutic modalities now being assessed for approval, would be very useful in clinical practice and could have a measurable impact in the care for patients with suspected food allergy and for food‐allergic patients.

## 3 WHAT IS THE RATIONALE FOR BASOPHIL TESTING IN IGE‐MEDIATED FOOD ALLERGY?

Basophils like mast cells express the tetrameric form of the high‐affinity IgE receptor and are thought to be directly involved in IgE‐mediated acute allergic reactions and anaphylaxis. In fact, fatal and near‐fatal cases of anaphylaxis have occurred without elevated tryptase, a mediator released by mast cells but not by basophils, raising the possibility that at least in some cases, reactions to foods may be driven primarily by basophils.^28^ Direct evidence of basophil activation during food allergy was recently provided by Commins et al.,^29^ who challenged patients with a form of allergy to red meat and showed that basophil activation coincided with the development of symptoms during the OFC in the majority of patients. In 9 of the 12 patients studied, no change in the tryptase level was observed at the different time points. This study reinforces the relevance of basophils in IgE‐mediated reactions to foods, including anaphylaxis.

Whether or not basophils play a unique role in vivo, BAT may offer several advantages over skin testing as a biomarker for disease, including the determination of a dose‐response for activation, the insensitivity to patient use of histamine blockers^30^ and the assessment of reactivity (when performed as is most common as a whole blood assay) in the presence of non‐IgE allergen‐specific antibodies (ie IgG, IgA), which may be important negative regulators of IgE‐mediated reactivity.^31^ BAT also has the advantages of being possible to perform in children with extensive eczema and of not being an in vivo test, thus with a higher safety profile.

The basophil activation test (BAT) assesses the expression of activation markers such as CD63 and CD203c on the surface of basophils by flow cytometry following stimulation with food allergens and controls.^32^, ^33^ The upregulation of CD63 or CD203c may not always correlate with the total histamine released, which has been suggested to be due to the fact that the two markers follow different pathways of basophil activation, with CD63 reflecting anaphylactic degranulation ad CD203c piecemeal degranulation.^34^ CD63 is highly relevant to IgE‐mediated allergic reactions as it directly correlates with histamine that is released and is in part responsible for patients' allergic symptoms.^35^ CD63 expression is inversely correlated with intracellular diaminoxidase, an enzyme localized to the same intracellular granules as histamine. Intracellular diaminoxidase is inversely correlated with the histamine that is released to the extracellular space.^36^ Studies assessing the expression of CD63 and diaminoxidase in the same samples further support the relevance of CD63 and the BAT in IgE‐mediated allergic reactions.^36^, ^37^

Different methods can be used to identify basophils in whole blood by flow cytometry^38^(Table 1) using different gating strategies, namely SSClow/IgE positive,^39^ SSClow/CD203c positive/CD123 positive/HLA‐DR negative,^4^, ^40^ CD45dim/CD123bright/HLA‐DR negative,^41^ SSClow/CCR3 positive^42^ or SSClow/CRTH2 positive/CD3 negative.^37^ The adopted gating strategy can have implications in the diagnostic performance of BAT. For instance, losing activated basophils to analyses with the adopted gating strategy can lead to false‐negative results; conversely, using a marker that is not specific for basophils can lead to the inclusion of additional cells with a loss of signal.^43^ Generally, the combination of a larger number of markers allows to select a purer population with the disadvantage of increasing the costs and laboriousness of the assay. Additional factors that can affect BAT results are the criteria to define the negative gate and the cut‐off for a positive BAT result.^44^

**Table 1:** Basophil identification markers

Marker	IgE	CD123	CCR3	CRTH2	CD203c
Synonym	‐	IL‐3Rα	CD193	CD294	Neural cell surface differentiation antigen
Function	Defence against helminths, type I hypersensitivity	Low‐affinity (α) subunit of IL‐3 receptor	Receptor for C‐C type chemokines	Receptor for prostaglandin D2	Unknown
Peripheral blood cells expressing the marker	Monocytes, dendritic cells, basophils, B cells and platelets	Basophils, monocytes, eosinophils, plasmacytoid dendritic cells, myeloid dendritic cells, and subsets of haematologic progenitor cells	Basophils, eosinophils, Th2 cells	Basophils, eosinophils, Th2 cells	Basophils
Markers to be used in combination	HLA‐DR^a^	HLA‐DR^a^	CD3^b^	CD3^b^	None^c^

a HLA‐DR is expressed on monocytes and dendritic cells allowing the distinction from basophils and eosinophils. The latter two types of cells have different size and granularity and can thus be distinguished using forward scatter and side scatter characteristics.

b CD3 is expressed on T cells and therefore allows the exclusion of this cell type when using CCR3 or CRTH2. The distinction between basophils and eosinophils can be carried out by size and granularity using forward scatter and side scatter.

c CD203c is specific for basophils and therefore can be used to identify basophils without other markers.

Abbreviations: CCR3, C‐C chemokine receptor type 3; CRTH2, chemoattractant receptor‐homologous molecule expressed on Th2 cells.

## 4 WHAT IS KNOWN ABOUT THE USE OF THE BASOPHIL ACTIVATION TEST TO DIAGNOSE IGE‐MEDIATED FOOD ALLERGY?

Various studies have assessed the utility of BAT to diagnose allergy to different foods since the first publication of the kind by Moneret‐Vautrin et al.^45^ Studied foods include cow's milk,^46^, ^47^ egg,^46^, ^48^ wheat,^49^, ^50^, ^51^, ^52^, ^53^ peanut,^4^, ^22^, ^48^, ^54^, ^55^ hazelnut,^56^, ^57^, ^58^, ^59^ shellfish^60^ and peach,^61^, ^62^, ^63^ apple,^64^ celery and carrot.^65^, ^66^ Generally, these studies showed that BAT has good sensitivity and specificity (Table 2), although some of them were small in size and not all used OFC as the comparator for BAT. The largest diagnostic study to date using BAT was a peanut study^4^ where a total of 169 patients were assessed for possible peanut allergy, including a primary population of 104 patients used to generate the optimal diagnostic cut‐offs, and a second population of 65 patients prospectively recruited used to externally validate the findings. In this study, BAT showed 98% sensitivity and 96% specificity in the primary population and 83% sensitivity and 100% specificity in the second population to diagnose peanut allergy.

**Table 2:** Examples of studies assessing the utility of the basophil activation test to diagnose food allergy using whole allergen extracts or single allergens

Food allergy	Food extract or allergen component	Study	Cut‐offs	Sensitivity	Specificity
Cow's milk allergy	Cow's milk extract	Rubio (2011)^68^	>6% CD63+	91%	90%
		Sato (2010)^46^	SI CD203c ≥1.9	89%	83%
	Casein	Sato (2010)^46^	SI CD203c ≥1.3^46^	67%	71%
Egg allergy	Ovalbumin	Ocmant (2009)^48^	≥5% CD63+	77% for CD63	100% for CD63
			SI CD203c ≥1.6	63% for CD203c	96% for CD203c
Baked egg allergy	Egg white extract	Sato (2010)^46^	SI CD203c ≥2.4	74%	62%
	Ovomucoid		SI CD203c ≥1.7	80%	73%
Raw egg allergy	Egg white extract	Sato (2010)^46^	SI CD203c ≥1.7	77%	63%
	Ovomucoid		SI CD203c ≥1.6	83%	83%
Wheat allergy	Wheat extract	Tokuda (2009)^50^	>11.1% CD203c+	86%	58%
	Omega‐5 gliadin (nTri a 19)		>14.4% CD203c+	86%	58%
	Omega‐5 gliadin (rTri a 19)		>7.9% CD203c+	83%	63%
Peanut allergy	Peanut extract	Santos (2014)^4^	≥4.78% CD63+	98%	96%
	Ara h 2	Glaumann (2012)^22^	ND	92%	77%
Hazelnut allergy	Hazelnut extract	Brandstrom (2015)^58^	CD‐sens >1.7	100%	97%
PFAS to hazelnut		Erdmann (2003)^65^	≥6.7% CD63+	85%	80%
Peach allergy	Peach extract	Gamboa (2007)^62^	>20% CD63+SI CD63 >2	87%	69%
	Pru p 3		>20% CD63+SI CD63 >2	77%	97%
PFAS to apple	Apple extract	Ebo (2005)^64^	≥17% CD63+	88%	75%
PFAS to carrot	Carrot	Erdmann (2003)^65^	≥8.9% CD63+^65^	85%	85%
PFAS to celery	Celery	Erdmann (2003)^65^	≥6.3% CD63+^65^	85%	80%

SI, stimulation index; PFAS, pollen‐food syndrome; ND, not determined.

The diagnostic performance of BAT is allergen specific and can vary with the allergen preparation used for cell stimulation in the assay. The use of individual allergens in the BAT has also been tested, for example using lipid transfer proteins (e.g., Pru p 3 from peach^62^ and Ara h 9 from peanut^39^), seed storage proteins (e.g., Ara h 1, Ara h 2, Ara h 3 and Ara h 6 from peanut^39^) and Bet v 1 homologues (e.g., Ara h 8 from peanut^67^). In these studies, BAT using single allergen components showed to be advantageous compared to BAT using food extracts to diagnose allergy to some foods (e.g., BAT to Pru p 3 to diagnose peach allergy) but not to others (e.g., BAT to casein to diagnose cow's milk allergy)—Table 2.

The enhanced specificity and retained sensitivity of BAT compared with SPT and specific IgE create the potential to reduce the number of patients referred for OFC compared to what is current practice. Santos et al.^4^ have documented a 67% reduction in the need for OFC in a peanut study. In a second population of patients, used for external validation of the test, the specificity of BAT reached 100%, meaning that a positive BAT would confirm the diagnosis of peanut allergy with a high degree of certainty. Therefore, the reduction in OFC was mostly a reduction in positive OFC, which would be most desirable in clinical practice.

## 5 WHAT IS KNOWN ABOUT THE USE OF THE BASOPHIL ACTIVATION TEST TO ASSESS PROGNOSIS IN IGE‐MEDIATED FOOD ALLERGY?

Oral food challenge is currently the method of choice not only to diagnose food allergy but also to assess response to treatment. All the current interventional studies for food allergy have as their primary end‐point the change in clinical sensitivity defined by the dose eliciting an objective reaction during a post‐treatment food challenge from that at baseline. Although safe when carried out in qualified centres, these OFCs for individuals known or strongly suspected to be allergic are often particularly stressful experiences for both patients and their families as well as for the clinical team performing the OFC. In addition, specific allergen immunotherapy‐based interventions, which are those that are closest to FDA approval and potential broad adoption in clinical practice, have been associated with variable outcomes. While some individuals appear to achieve a sustained long‐term benefit without need of continuous treatment, others appear to have rapidly waning benefit. Current testing does not distinguish these individuals raising the potential need for periodically repeated OFC unless other biomarkers can be identified.

The BAT has been used to monitor the acquisition of oral tolerance to foods over time, either naturally or under immunomodulatory interventions. Wanich et al.^40^ showed that basophil reactivity following stimulation with a cow's milk extract reflected different phenotypes of cow's milk allergy, with patients that tolerated heated milk showing a degree of basophil reactivity that was intermediate between that of patients allergic to all forms of cow's milk and patients that had outgrown their cow's milk allergy. In another study, BAT to cow's milk showed to be useful in identifying patients who had resolved their cow's milk allergy.^68^ Basophil activation is modified during allergen‐specific immunotherapy and has shown to be reduced in patients submitted to oral immunotherapy to foods such as cow's milk, peanut and egg.^69^, ^70^, ^71^, ^72^ Basophil suppression during desensitization can be observed not only following stimulation with the implicated allergen but also with a bystander allergen and an IgE‐mediated positive control.^73^ It remains to be seen whether basophil suppression persists following discontinuation of allergen‐specific immunotherapy. Basophil activation in food‐allergic patients is reduced during treatment with omalizumab but increases after cessation of treatment.^74^ The reduction in basophil activation following treatment with omalizumab seems to be dependent on low specific/total IgE ratios and effects on the intrinsic basophil reactivity potentially mediated by a variety of mechanisms.^75^, ^76^, ^77^, ^78^

The results of the BAT have been associated with the severity and the threshold of allergic reactions during the OFC. The proportion of activated basophils in response to allergen in vitro, so‐called basophil reactivity, has been directly correlated with the severity of symptoms experienced during OFC in studies of mostly peanut and cow's milk‐allergic patients.^68^, ^79^, ^80^ Measures of in vitro basophil sensitivity, such as “CD‐sens,” in one study^79^ and the ratio between activated basophils following stimulation with allergen and an IgE‐mediated control in another study^68^ have been correlated with the threshold of reactivity during OFC. These data suggest that BAT can provide information about the severity and the threshold of allergic reactions that, in addition to other clinical characteristics of the patients that have been identified as risk factors (e.g., persistent asthma), might enable the clinician to identify high‐risk allergic patients who require closer follow‐up and more intensified education.

Among the limitations of BAT for routine use are the proportion of patients with non‐responder basophils (ie basophils selectively unresponsive to FcERI‐mediated signalling, which has been reported in as many as 17% of individuals^48^), the fact that the test requires fresh blood (<24 hours since blood collection^81^) to run, the need for standardized allergens whose optimal concentration is variable depending on the specific allergen and the characteristics of the extract. Furthermore, BAT requires expertise to perform a flow cytometry‐based assay that is not currently automated and to subsequently analyse the data.

## 6 WHAT WOULD BE THE VALUE OF USING BAT IN CLINICAL PRACTICE?

The high specificity of BAT confers its greatest advantage compared with tests that are currently used in clinical practice, such as SPT and specific IgE.^4^, ^59^ For the foods where component testing offers added value compared to using whole allergen extracts, enhanced specificity can be obtained with specific IgE to component allergens; however, cases remain where specific IgE to individual components is not enough to reach a clear definitive diagnosis of food allergy. An example is Ara h 2‐specific IgE which has shown high accuracy to diagnose peanut allergy and higher than that of specific IgE to peanut but not than that of BAT to peanut,^4^, ^22^ indicating that some patients may benefit from BAT to peanut in addition to or as an alternative to IgE to Ara h 2.^4^ Another example is the Cor a 9‐ and Cor a 14‐specific IgE which have shown to be more accurate than specific IgE to hazelnut in the diagnosis of hazelnut allergy^12^, ^13^; however, in a study where these two hazelnut components were tested together with BAT to hazelnut, BAT to hazelnut showed higher specificity (97%) than specific IgE to Cor a 14 (94%) and specific IgE to Cor a 9 (72%).^58^ The improved diagnostic performance of BAT compared to specific IgE to allergen components which themselves have shown to have very good diagnostic performance is probably related to the fact that BAT is a functional assay, whose results depend not only on the amount of IgE but also on other characteristics of IgE (such as affinity and clonality) and possibly of antibodies of other isotypes (such as IgG4) which together are responsible for the ability of allergen to trigger effector cell activation.^31^

Basophil activation test can be performed using single allergen components, which for some foods can be more accurate than using allergen extracts in the BAT (Table 2). For example, BAT to ovomucoid and BAT to Pru p 3 showed improved diagnostic accuracy compared to BAT to egg white and BAT to peach to diagnose egg allergy (both baked and raw egg allergies) and peach allergy, respectively.^46^, ^62^ The use of single allergens has, however, the disadvantage of missing the contribution of minor allergens that are clinically relevant for some patients and of missing the combined effect of multiple allergens to which polysensitized patients produce IgE and which may increase the degree of basophil activation detected in the BAT.

**Figure 1:**
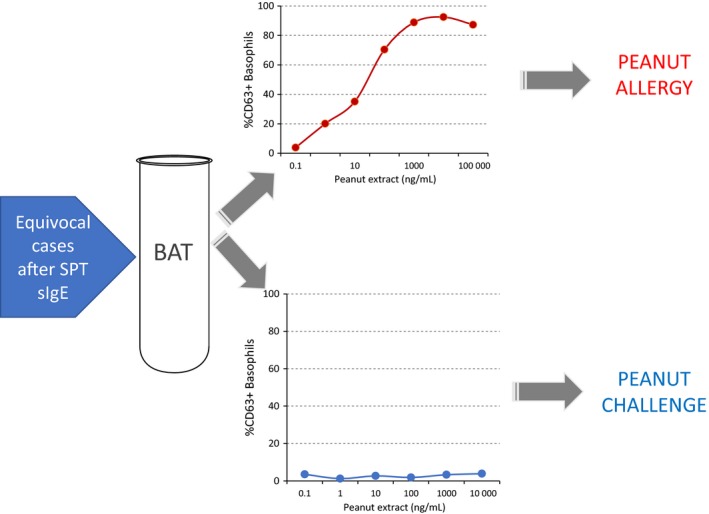
Proposed approach to using BAT to diagnose peanut^4^ and possibly other food allergies. BAT is performed in patients with equivocal results of skin prick test (SPT) and/or specific IgE. Patients with a positive BAT are advised to avoid peanut and patients with a negative BAT or non‐responder basophils are offered an oral food challenge

Due to its high specificity, which reaches 100% in some studies,^4^, ^48^ a positive BAT allows confirming the diagnosis of IgE‐mediated food allergy with a high degree of certainty. Given the practical implications involved in the performance of the BAT and the fact that in some patients an allergy‐focused clinical history together with the documentation of specific IgE to extracts or components is sufficient to confirm or exclude the diagnosis of IgE‐mediated food allergy, the BAT is probably a test that is worth doing in selected patients with suspected food allergy. It could thus be used as a second step in the diagnostic process, in patients that would otherwise be referred for an OFC following the appropriate clinical assessment and the performance of SPT and/or specific IgE. This two‐step approach has the advantage of requiring the performance of a smaller number of BAT. Patients with a positive BAT would see their OFC obviated and would be spared from experiencing an acute allergic reaction during the OFC. Patients with a negative BAT or non‐responder basophils would need to be referred for an OFC (Figure 1). For example, in a peanut allergy study,^4^ using this two‐step approach, BAT would need to be performed in less than 25% of assessed patients and allowed for a 67% reduction in peanut OFC.

Being a blood test that requires a small volume of blood (about 1 mL) and therefore is minimally invasive, BAT has a good chance to be well accepted by patients and families. However, because it requires fresh blood, it cannot be performed using stored frozen samples of blood and patients need to book an appointment to have the BAT done. To reduce the costs and the number of blood draws, it may be preferable to perform BAT following SPT, if available. SPT provides an immediate result and in cases for which the combination of history and SPT are equivocal, blood for BAT (and specific IgE) could be collected on the same day as the clinical appointment.

With the recent change in the guidelines for the prevention of peanut allergy,^82^, ^83^ recommending introduction of peanut in the first year of life and between the age of 4 and 6 months in high‐risk infants, a potential application of the BAT is the assessment of high‐risk infants before introduction of peanut. BAT has shown to be more accurate than SPT and specific IgE to diagnose peanut allergy in children including children in the first year of life.^4^ Using BAT in peanut‐sensitized infants could reduce the number of positive OFC. Because acute allergic reactions and anaphylaxis are challenging to diagnose and to treat in infancy, BAT could reduce the number of adverse events and enhance patients' comfort and safety at this young age. Recent guidelines^82^ contemplate only SPT and specific IgE in the assessment of patients prior to the introduction of peanut, as BAT is not yet established clinically.

## 7 WHAT ARE THE NEXT STEPS TO BRING THE BAT TO THE CLINIC?

**Figure 2:**
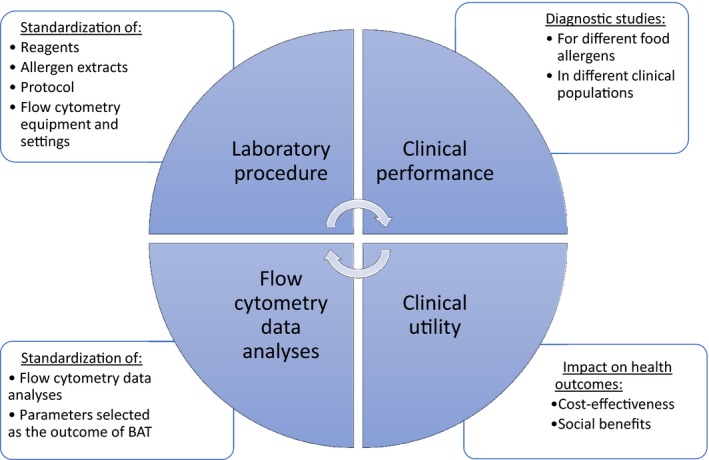
Road map to bring BAT from the research laboratory to clinical practice

The performance of BAT to diagnose IgE‐mediated food allergies can vary with different factors, some related to the study population, some related to the study design, some to the laboratory procedure selected for the BAT and some to the analyses of flow cytometry data.^32^, ^84^ The clinical application of BAT would require, on the one hand, the standardization of the laboratory procedure and data analyses and, on the other hand, the clinical validation of the test, that could enable regulatory approval and eventual incorporation into guidelines, provided continued quality control and sufficient scientific evidence to support its clinical use was available (Figure 2).

Currently, significant heterogeneity exists in published studies with regard to most of these aspects.^32^ In terms of laboratory procedure, various methods have been developed in‐house and kits are also commercially available. The latter may offer a greater sense of security to less experienced users and standardization across sites; however, the procedure does not differ significantly compared to in‐house methods and has the disadvantage of not disclosing all information about the reagents included in the kit. The methods used for BAT can vary with regard to sample anti‐coagulation, the stimulation buffer used—especially the presence and concentration of IL‐3, the time allowed between blood collection and performance of the assay, the allergens used for cell stimulation, the antibodies used for cell staining, whether stimulation and staining are done simultaneously or as separate steps, buffer used for erythrocyte lysis and additional washing steps.^22^, ^35^, ^38^, ^81^, ^85^, ^86^, ^87^, ^88^ All these steps can affect the diagnostic performance of BAT. The extracts or the allergens used for cell stimulation can also impact the results and vary over time if there is no standardization in place. In this regard, recombinant allergens offer the highest stability and consistency, compared to purified allergens or allergen extracts, but the potential disadvantage of failing to account for the contributions of reactivity to minor allergens.

The methods used for flow cytometry data collection (e.g., flow cytometry instrument and settings used over time) can also significantly impact on the BAT results^89^ and must be standardized and described.^90^ In addition, the criteria adopted for the analyses when performed “manually,” as is still often the case for flow cytometry data, are subject to significant subjectivity and poor reproducibility. Automated analysis platforms and methods are being developed to meet the need for more standardized data analyses.^91^, ^92^ Automated analyses approaches have significant advantages of being more time‐efficient, reproducible and high‐throughput; however, they may be more sensitive to the acquisition of high‐quality flow cytometry data (not necessarily a bad thing) and may be challenging to generalize to multiple BAT assays that may employ different antigens, equipment and other sources of variation. Finally, the “read‐out” parameter(s) selected to express the results of BAT, and the method used to calculate it is another important aspect that needs to be standardized to ensure consistency and reproducibility of BAT results. Ideally, multi‐centre studies should be performed to assess the reproducibility and variability of the test procedure.

As with any allergy test, BAT requires clinical validation through the performance of diagnostic studies in different food allergies, as identified diagnostic decision levels are allergen specific. They are also specific to the population where they are generated, as they depend on the prevalence of food allergy in the population and can vary with the geographical location and other factors that can affect the clinical phenotype of patients being assessed for suspected food allergy. While the identified diagnostic cut‐offs can be extrapolated to a population with similar characteristics to the one where they were generated, the application of the test to a different patient population would in principle require a separate clinical validation.

Before regulatory approval can be granted, continuous quality control needs to be in place and evidence for cost‐effectiveness and beneficial effects on health and social outcomes should be gathered (Figure 2). Although BAT is likely much cheaper and certainly safer than OFC, it is probably more expensive than SPT or specific IgE. Formal cost‐effectiveness studies would be warranted.

Basophil activation test is a novel test which is quite distinct from standard allergy tests currently in use. Its incorporation in clinical practice requires dissemination of information about the test, namely its methodology and interpretation of results, so that an improved understanding of the assay can enhance confidence in its clinical use. Once the BAT is being used in clinical practice, its impact on patients' health outcomes and on the decision‐making process involved in the referral for OFC and in the confirmation or exclusion of the diagnosis of food allergy should be assessed to sustain its clinical use. Another important aspect that should be explored in future research is the need for more sophisticated multi‐dimensional diagnostic algorithms using large sets of clinical and laboratory values to determine a comprehensive “post‐test diagnostic probability.” Such analyses could provide valuable information on the combination of diagnostic tests for optimal food allergy diagnosis.

## 8 CONCLUSIONS

BAT is sensitive and more specific than standard testing and has the potential to reduce the number of OFC currently needed to diagnose IgE‐mediated food allergy as well as to monitor clinical response to treatment and possible resolution of food allergy. Because of the better specificity, the most of the OFC avoided would be those that are positive, which would improve patient safety and comfort. However, in order to achieve a widespread adoption of BAT in clinical practice, it is necessary to achieve standardization of the laboratory procedure and data analyses and more rigorous validation. Finally, a comprehensive assessment of the impact of BAT on health and social outcomes and its cost‐effectiveness would be warranted.

## CONFLICT OF INTEREST

The authors declare no conflict of interest.
